# Inhibition of Return Decreases Early Audiovisual Integration: An Event-Related Potential Study

**DOI:** 10.3389/fnhum.2021.712958

**Published:** 2021-10-06

**Authors:** Xing Peng, Xiaoyu Tang, Hao Jiang, Aijun Wang, Ming Zhang, Ruosong Chang

**Affiliations:** ^1^Institute of Aviation Human Factors and Ergonomics, College of Flight Technology, Civil Aviation Flight University of China, Guanghan, China; ^2^School of Psychology, Liaoning Collaborative Innovation Center of Children and Adolescents Healthy Personality Assessment and Cultivation, Liaoning Normal University, Dalian, China; ^3^Department of Psychology, Soochow University, Suzhou, China

**Keywords:** audiovisual integration, inhibition of return, exogenous spatial attention, cue-target paradigm, event-related potentials

## Abstract

Previous behavioral studies have found that inhibition of return decreases the audiovisual integration, while the underlying neural mechanisms are unknown. The current work utilized the high temporal resolution of event-related potentials (ERPs) to investigate how audiovisual integration would be modulated by inhibition of return. We employed the cue-target paradigm and manipulated the target type and cue validity. Participants were required to perform the task of detection of visual (V), auditory (A), or audiovisual (AV) targets shown in the identical (valid cue) or opposed (invalid cue) side to be the preceding exogenous cue. The neural activities between AV targets and the sum of the A and V targets were compared, and their differences were calculated to present the audiovisual integration effect in different cue validity conditions (valid, invalid). The ERPs results showed that a significant super-additive audiovisual integration effect was observed on the P70 (60∼90 ms, frontal-central) only under the invalid cue condition. The significant audiovisual integration effects were observed on the N1 or P2 components (N1, 120∼180 ms, frontal-central-parietal; P2, 200∼260 ms, frontal-central-parietal) in both valid cue as well as invalid cue condition. And there were no significant differences on the later components between invalid cue and valid cue. The result offers the first neural demonstration that inhibition of return modulates the early audiovisual integration process.

## Introduction

In everyday life, human perceptual systems are frequently overwhelmed by inputs from multiple sensory systems at once. Multisensory integration mechanisms, such as the mechanism responsible for audiovisual integration (AVI) can integrate information from multiple sensory modalities into a unified and meaningful representation (Stein and Meredith, 1993; Molholm et al., 2002; Talsma et al., 2010b; Tang et al., 2016). Similarly, attention can also help the brain select useful stimuli from various sensory modalities (Corbetta and Shulman, 2002; Petersen and Posner, 2012; Tang et al., 2016). Audiovisual integration and attention are two important mechanisms that help to combine and process information from different sensory modalities. We previously developed a structure illustrating the interactive processes of audiovisual integration and endogenous or exogenous attention (see Tang et al., 2016, for a review). On one hand, audiovisual integration exerts not only bottom-up but also top-down control over attention. On the other hand, attention is a mechanism critically impacting audiovisual integration processing. Researchers have already investigated the influence of endogenous attention (via instructions to make subjects attend to one/two locations or modalities) on audiovisual integration (Talsma and Woldorff, 2005; Talsma et al., 2007, 2010a; Mikhail et al., 2013). Studies using event-related potentials (ERPs) have shown that endogenous spatial attention enhances audiovisual integration within 100 ms of stimulation (Talsma and Woldorff, 2005; Talsma et al., 2007).

However, few studies to date have investigated the relationship between exogenous spatial attention (due to salience of a stimulus) and audiovisual integration. Van der Stoep et al. (2015, 2017) investigated this relationship in behavioral studies based on an exogenous cue-target paradigm − a classical paradigm for studying attention. In this paradigm, an abrupt peripheral stimulus (i.e., an exogenous cue) is presented to the left or right of fixation. After a brief cue-target interval (stimulus-onset asynchrony, SOA), participants are asked to press a button to respond to a target which appears at either the identical location (valid cue) or the opposite side of visual fixation (invalid cue). When the SOA is less than about 250 ms, responses to validly cued targets are faster than those to invalidly cued targets. This is called the “facilitation effect.” By contrast, reaction times to validly cued targets are slower than those to invalidly cued targets when the SOA is longer than 300 ms. This is termed the “inhibition of return” or “IOR” effect (Posner et al., 1985).

Van der Stoep et al. (2017) employed a longer SOA (350–450 ms) to examine the impacts of the IOR effects induced by exogenous visual cues on audiovisual integration. Behavioral results showed that IOR effects reduced the audiovisual integration, and the audiovisual integration effect at the valid cue location was noticeably smaller in contrast to that at invalid cue location. The assumption of differences in unimodal signal strength has been put forward to explain this result. Specifically, the benefit achieved by audiovisual integration is most prominent if one modality shows dominance performance in various modalities (Ernst and Banks, 2002; Otto et al., 2013). In other words, when the difference in signal strength between different sensory modalities is larger, the AVI effects will be smaller. On the contrary, when the signal strengths between different sensory modalities are more similar, AVI effects will be larger. In this study, exogenous cue induced an IOR effect on visual targets but not on auditory targets. Therefore, when the RT-differences between A and V are larger with valid cues, the differences of signal strength will also be large, which further led to smaller audiovisual integration effects at the valid cue location.

It is worth noting that the assumption of differences in unimodal signal strength is based on behavioral results. The neural mechanisms underlying this modulation effect are unknown. As ERPs can reveal the time-course of processing through several phases of audiovisual integration, we will use this technique to explore the neural underpinnings of modulation of audiovisual integration by IOR. In our study, we apply the exogenous cue-target paradigm and manipulate factors of target type (audiovisual, visual, auditory) as well as cue validity (invalid, valid). By exploiting ERPs’ high temporal resolution, the observation of which stage(s) of audiovisual integration processes are under the influence of IOR can be achieved.

## Materials and Methods

### Participants

We determined a suitable sample size based on the previous behavioral study (Van der Stoep et al., 2017) and the G^∗^Power toolbox (Faul et al., 2007). For the suggested 95% statistical power at α = 0.05, and an effect size of 0.8, we determined that the appropriate sample size was no fewer than 12 participants. Therefore, 21 undergraduates were recruited with an age range of 19∼24 years old, *M* = 20.48, and *SD* = 1.2. Written informed consent was offered by all subjects engaged.

Participants were all right-handed and reported normal or corrected-to-normal vision. No participant reported any history of neurological or psychiatric disorders.

### Stimuli and Procedure

**FIGURE 1:**
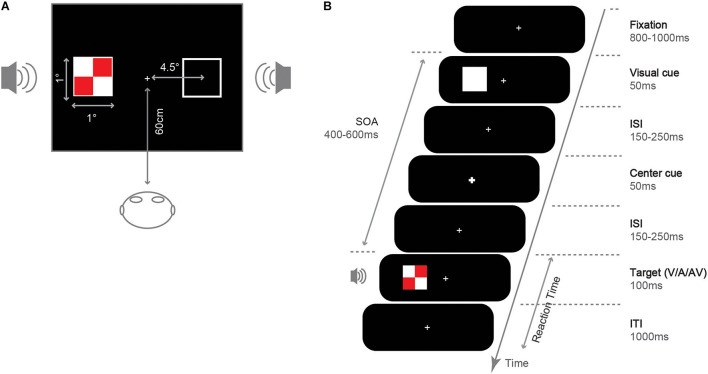
Illustration for the stimuli and experiment. **(A)** Size and position. **(B)** Sequence of event and duration under the valid cue condition.

We presented experimental stimuli on an LCD screen (resolution 1024 × 768 pixels, 100 Hz refresh rate). The experimental procedure was programmed using E-prime 1.1 software. We presented all stimuli on a black background (0.4 cd/m^2^). Participants completed the experiment in a dark, sound-attenuated room and sat 60 cm away from the display (see Figure 1A). As shown in Figure 1B, in the fixation screen, a white (RGB: 255, 255, 255; 155.2 cd/m^2^) fixation cross (0.05° × 0.05° of the visual angle) was presented at the center of the screen. Then, an uninformative white square was presented randomly at the left or right side of the fixation (1° × 1°, 4.5° eccentricity) to capture attention in that location as an exogenous cue. In the central cue screen, the central fixation became larger and thicker (0.1° × 0.1°) to reorient attention at central location. The central cue was designed to facilitate the IOR effect, even under small SOA and cross-modality conditions (Pratt and Fischer, 2002; Prime and Jolicoeur, 2010). In the target screen, a visual, auditory, or audiovisual target was presented. Visual target stimuli were red (RGB: 255, 0, 0; 27.5 cd/m^2^) and white checkerboard (length: 1° × 1°, subtending a visual angle of 0.8° × 0.8°). Auditory target stimuli were sinusoidal tone of 1,000 Hz (65 dB, 100 ms, 10 ms rising and falling time), created and processed using the software Sound Engine 2.0, and played through two speakers placed on the right and left sides. Audiovisual targets were the synchronous presentation of the visual and the auditory stimuli, and the audiovisual targets always appeared on the same side.

### Procedure and Task

We manipulated 2 factors of target type (V, AV, A) and cue validity (valid, invalid). In valid cue trials, the cue and the target showed up in the identical location. In invalid cue trials, they showed up in opposed locations. The amount of valid and invalid cue trials was the same. The numbers of V, AV, and A trials were also the same. The participants were asked to look at the central fixation throughout the experiment. Following the practice block (56 trials), the participant finished eight experimental blocks of 1120 trials in total. In the experiment, target stimuli were presented in 6/7 of the trials. The other 1/7 of the trials were catch trials (no target presented). The participants received the instruction for pressing button “B” to respond to the target stimulus (A, V, or AV) at any possible location as quickly and accurately as possible. As no target was presented in the catch trials, only the trials containing the target stimuli (6/7 of all trials) were analyzed, i.e., a total of 960 trials. The 160 trials for each condition were randomly presented. After each block, there was feedback on the accuracy of the previous block. Participants can take a break between blocks. The entire experiment lasted for approximately 60 min.

The procedure for a single trial was shown in Figure 1B: each trial started with the presentation of a fixation cross, lasting for 800∼1,000 ms. Then, a white square (exogenous spatial cue) was shown on the left or right side of the screen and lasted for 50 ms. After a random interval of 150∼250 ms, the fixation became larger and thicker (central visual cue) with a 50 ms duration. After a random interval of 150∼250 ms since the offset of the central cue, a target stimulus (V/A/AV) was presented on the screen’s right or left side with a 100 ms duration. Thus, the SOA of the target and the exogenous spatial cue was 400∼600 ms. Finally, the fixation was presented for 1,000 ms, during which participants could respond via button press.

### Data Recording and Analyses

#### Behavioral Measure

We calculated the average accuracy (ACC) and reaction time (RT) for each participant. The ACC for each participant was higher than 95%. Thus, the accuracy data were not analyzed further, given that the ACCs were close to the ceiling. A comparison was drawn for the RT with the 3 (target type: A, V, or AV) × 2 (cue validity: valid, invalid) repeated measure ANOVA.

#### Event-Related Potentials Measures

We employed a 32-channel EEG system (Brain Products, Brain Vision Recorder 2.0) with electrodes positioned according to the International 10-20 system using an electrode cap (acti CHamp, Inc.). The following electrodes were used: Fp1, Fp2, F3, F4, F7, F8, Fz, FC1, FC2, FC5, FC6, Cz, C3, C4, T7, T8, CP1, CP2, CP5, CP6, TP9, TP10, P3, P4, Pz, P7, P8, O1, Oz, O2, the reference and ground electrodes. The reference electrode was located on the left earlobe, and the ground on the frontal midline. Horizontal eye movements were recorded from the outer canthus of the left eye, and vertical eye movements and blinks were recorded from an electrode placed 1.5 cm below the left eye. Impedance on all electrodes was below 5 kΩ. We digitized EEG using a sampling frequency of 500 Hz. ERP data were analyzed off-line with the use of Brain Vision Analyzer (version 2.0, Germany). The data were filtered by using a band-pass filter retaining frequencies between 0.1 and 30 Hz (slope = 24d B/octave), and then re-referenced to the average of the two earlobes. The continuous EEG signal fell to epochs from −100 to 500 ms. Baseline correction was conducted for the data from −100 to 0 ms. Artifact rejection was performed using a semi-automated procedure to remove epochs that contained eye movements and blinks from EEG. Also, signal artifacts were detected as amplitudes exceeding ± 80μV, differences beyond 100 μV within a 200 ms interval.

According to the previous research, exogenous attention effects (IOR) on the audiovisual object ERPs consisted mainly of P1 (60∼100 ms), N1(120∼180 ms), P2 (200∼260 ms). The P1/N1 components were measured at lateral frontal electrodes (F7/F8) (Pierce et al., 2018), P2 component was measured at the posterior parietal cortex (Pz/P3/P4) (Li et al., 2018). The audiovisual integration was investigated by the [AV − (A + V)] equation. ERPs from the unisensory auditory (A) and visual (V) stimuli were summed and compared with the ERPs elicited by AV stimuli. The mean amplitude of early P70 (60∼90 ms) was measured at FCz, FC1, FC2, Fz, Cz, Pz, P3, P4 electrodes, N1(120∼180 ms), P2 (200∼260 ms)were measured at FCz, FC1, FC2, Fz, F3, F4, Cz, C3, C4, Pz, P3, P4 electrodes (Talsma and Woldorff, 2005; Talsma et al., 2007). We only compared the ERP results of audiovisual targets at different cue validity conditions because the simultaneous presentation of visual and auditory stimuli can produce audiovisual integration. Besides, the reduction of IOR in behavioral data mainly occurs in audiovisual targets.

In these time windows, the mean amplitude data were analyzed using repeated-measures ANOVA with factors of integration (AV, summed unisensory (A + V) ERPs) and cue validity (valid cue or invalid cue). The Greenhouse-Geisser epsilon or Bonferroni correction was used for non-sphericity or *post hoc* comparisons. All statistical levels (i.e., α level) were set to 0.05. The effect size of Cohen’s *d* or partial eta-squared ($\eta_{p}^{2}$) was calculated for mean comparisons or ANOVA, respectively.

## Results

### Behavioral Data

A 3 (target type: A, V, and AV) × 2 (cue validity: valid cue, invalid cue) repeated-measures ANOVA was applied on reaction times. Reaction times (RTs) were excluded based on the following criteria: RT with the incorrect response, RT with no response, RT less than 100 ms or longer than 1,000 ms. The discarded data were 3% of the total.

The main effect of the target type was significant [*F* (2, 40) = 67.66, *p* < 0.001, η_*p*_^2^ = 0.77], which was driven by AV targets (330 ms) being faster than A (352 ms) and the V (390 ms) targets. The main effect of cue validity was significant [*F* (1, 20) = 80.62, *p* < 0.001, η_*p*_^2^ = 0.80]. The results showed that the responses under the invalid cue condition (348 ms) were faster than those in the valid cue condition (366 ms), which suggested that an IOR occurred. Additionally, the interaction between the target type and cue validity was also significant [*F* (2, 40) = 30.55, *p* < 0.001, η_*p*_^2^ = 0.60]. The IOR effect was significant for the V (31 ms, *t* (20) = 8.91, *p* < 0.001, *d* = 0.34), AV (12 ms, *t* (20) = 5.11, *p* < 0.001, *d* = 0.17) and A targets (10 ms, *t* (20) = 3.56, *p* < 0.001, *d* = 0.13).

Table 1 shows the results of planned comparisons analyzed using *t*-tests. The IOR effect of the V targets was even larger than the IOR effect of the AV targets [31 ms vs. 12 ms; *t* (20) = 6.12, *p* < 0.001].

**TABLE 1:** Comparison of the IOR effects (ms) and contrasts between different conditions adopting a *t* test (sig. two-tailed, 95% confidence interval, CI).

	*M* (ms)	95% CI		*t*	*p*
		Lower (ms)	Upper (ms)		
**IOR**					
A	9.57	4.51	14.63	3.95	0.001
AV	12.10	7.54	16.65	5.54	0.000
V	30.86	24.39	37.32	9.96	0.000
**IOR Contrasts**					
AV vs. V	−18.76	−25.15	−12.37	6.12	0.000
V vs. A	21.29	27.50	15.07	7.14	0.000
AV vs. A	2.52	8.53	3.48	0.88	0.390

### Event-Related Potential Data

#### Valid Audiovisual vs. Invalid Audiovisual

**FIGURE 2:**
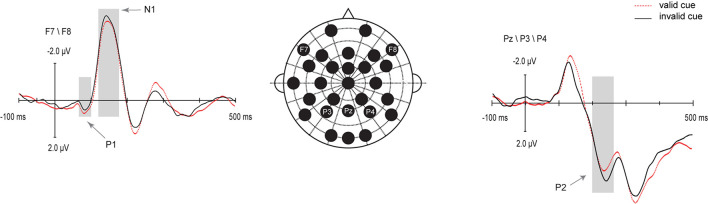
Grand average ERPs from audiovisual stimuli in valid cue (red dotted line) and invalid cue (black solid line) conditions on the analyzed electrodes.

IOR effects on audiovisual ERPs were visible mainly on the bilateral frontal P1 and N1 components, and the P2 component over the posterior parietal cortex. The selected electrodes for the analysis in this study are represented on the map in Figure 2. These amplitudes were subjected to paired samples *t*-test (valid, invalid).

The P1 and N1 effects were statistically tested by determining the mean amplitude on channels F7 and F8. As can be seen in Figure 2, the P1 was significantly larger for valid cue (0.79 μV) as compared with that for invalid cue (0.50 μV) objects, *t* (20) = 2.41, *p* = 0.026, *d* = 0.57. The N1 effect was statistically tested by the windows around 120∼180 ms which was significantly larger for invalid cue (−4.40 μV) as compared with that for valid cue (−4.04 μV) objects, *t* (20) = 2.45, *p* = 0.023, *d* = 0.22. The P2 effect was statistically tested on channels P3, P4, and Pz, which was significantly larger for invalid cue (3.69μV) as compared with that for invalid cue (3 μV) objects, *t* (20) = −5.38, *p* < 0.001.

#### Early P70 Modulations of Integration and Inhibition of Return Analyses

The interaction between IOR and audiovisual integration was determined by measuring the P70 amplitudes at FCz, FC1, FC2, Fz, Cz, Pz, P3, and P4 electrodes. The amplitude measures were submitted to ANOVA with the within-subject factors stimulus type (AV vs. [A + V]) and cue validity (valid vs. invalid).

**FIGURE 3:**
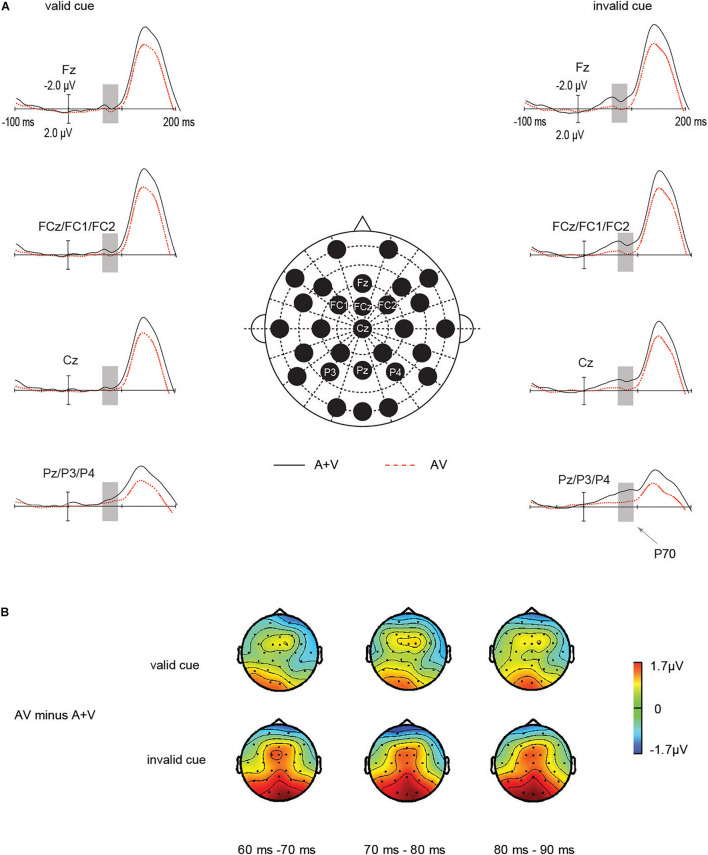
Audiovisual integration by IOR interactions on the fronto-central P70 components. The P70 components for the audiovisual stimuli were noticeably greater under the invalid cue condition as compared with that for the total unisensory response, whereas this study did not find such an effect in the valid cue. **(A)** Grand average ERPs of differences between AV (red dotted line) and A + V (black solid line) conditions. **(B)** The scalp topographies of the P70 components of AV minus A + V condition.

**ERP waveforms:** As suggested in Figure 3A, the main effect of cue validity was significant in the ANOVA [*F*(1, 20) = 6.23, *p* = 0.02, η_*p*_^2^ = 0.2]. P70 amplitude in invalid cue condition (−0.76 μV) was larger than in valid cue condition (−0.30 μV, *p* = 0.02). And the main effect of target type was not significant [*F* (1, 20) = 3.27, *p* = 0.08, η_*p*_^2^ = 0.14]. Importantly, the amplitudes of the early P70 components of unisensory and audiovisual stimuli were highly determined by IOR, which was expressed in a significant interaction between the stimulus type (AV vs. [A + V]) and IOR [*F* (1, 20) = 4.43, *p* = 0.048, η_*p*_^2^ = 0.18]. Under the invalid cue condition, the P70 amplitude from the sum of the unisensory auditory and visual stimuli activity (−1.25μV) was significantly larger than audiovisual stimuli (−0.28 μV), *t* (20) = 2.46, *p* = 0.023, *d* = 0.43. In contrast, in the valid cue condition, the P70 amplitudes was not significant between the audiovisual ERPs (−0.16 μV) and the summated unisensory ERPs (−0.4 5μV), *t* (20) = 0.78, *p* = 0.44. In summary, the interactive processes of audiovisual integration and IOR on P70 amplitude were found only under the invalid cue condition. These results indicated that the audiovisual integration was significantly larger for the invalid cue as compared with valid cue targets.

**Scalp topographies:** To further assess whether the modulation under the observation referred to the manipulation of P70, we investigated scalp topography exhibited by the mentioned effect for valid cue as well as invalid cue conditions in a separate manner. The mentioned investigation was carried out based on topography-normalized voltage (McCarthy and Wood, 1985) according to one subdivided set of frontal-central-parietal channel, which acted as the input for within-subject ANOVA. Figure 3B shows the early fronto-central-parietal P70 waveforms in invalid cue, in comparison with valid cue.

#### Audiovisual Integration Effects

**FIGURE 4:**
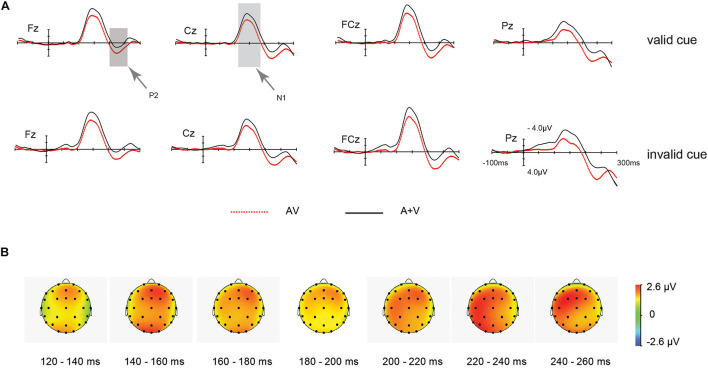
ERP waveform for the unisensory and multisensory (AV) late processing. **(A)** Grand average ERPs of differences between AV (red dotted line) and A + V (black solid line) conditions for valid and invalid cue stimuli. **(B)** Scalp topographies from 120 to 260 ms after the onset of the stimulus for the different waves of the multisensory (AV) and the summed (A + V) ERP responses, indicating multisensory integration effect for valid and invalid cue.

**ERP waveforms:** As shown in Figure 4, Subsequent audiovisual integration effects were observed on the fronto-central-parietal N1(120∼180 ms) and P2 (200∼260 ms) components that followed the P70. Besides, a 2 (cue validity: valid, invalid) × 2 (stimulus type: AV, A + V) repeated measures ANOVA was applied on the amplitude of (AV and [A + V]) again.

As for N1 effect, the main effect of cue validity was non-significant in the ANOVA [*F* (1, 20) = 0.33, *p* = 0.57, η_*p*_^2^ = 0.16]. And the main effect of target type was significant [*F* (1, 20) = 15.95, *p* = 0.001, η_*p*_^2^ = 0.44]. N1 amplitude of A + V (−7.16μV) was larger than AV condition (−5.46 μV). Nevertheless, the interaction between stimulus type (AV vs. [A + V]) and IOR was non-significant [*F* (1, 20) = 0.028, *p* = 0.87, η_*p*_^2^ = 0.101].

As for P2 effect, the main effect exerted by cue validity was non-significant in the ANOVA [*F* (1, 20) = 1.08, *p* = 0.31, η_*p*_^2^ = 0.05]. The main effect of target modality was significant [*F*(1, 20) = 10.51, *p* = 0.004, η_*p*_^2^ = 0.34]. P2 amplitude of AV (3.23 μV) was larger than A + V (1.28 μV) condition. Also, the interaction between the two factors was non-significant [*F*(1, 20) = 0.015, *p* = 0.91, η_*p*_^2^ = 0.001].

**Scalp topographies:** The audiovisual integration scalp topography of N1 or P2 received the test through the computation of the unisensory and audiovisual processing’s mean amplitude, across the 120∼260 ms time window. Furthermore, this interaction of valid and invalid cue was not significant (*F* < 1).

## Discussion

### Inhibition of Return of Audiovisual Stimuli

Behavioral results revealed significant IOR effects for visual, auditory, and audiovisual target stimuli. While the IOR effect for audiovisual targets following visual cues conforms to some existing research (Tang et al., 2019), other authors have not observed this effect (Van der Stoep et al., 2017).

Notably, we found the IOR effect elicited by audiovisual targets has been observed to decrease when paying attention to both visual and auditory modalities. According to this result, paying attention to multiple modalities simultaneously could modulate audiovisual integration (Talsma and Woldorff, 2005; Talsma et al., 2007). Specifically, based on the perceptual/attentional assumption, the biased attention causes smaller attention resource to the valid cue location and subsequently damages the perceptual processing of targets at the valid cue location; as a result, the manual response to a target presented at the valid cue location is decelerated (McDonald et al., 1999; Prime and Ward, 2004; Prime et al., 2006; Satel et al., 2013). Additionally, the auditory signal and concurrent visual event are capable of increasing visual brightness perceived (Stein et al., 1996), as well as decreasing visual contrast threshold (Lippert et al., 2007; Noesselt et al., 2010). In other words, the attended auditory stimulus is able to undergo the integration with a simultaneous visual stimulus, thereby enhancing the perceptual salience of a simultaneous visual stimulus. The reduced salience of a valid cue location (due to IOR) (Klein, 1988; Prime et al., 2006; Koningsbruggen et al., 2010) is offset by the increased perceptual salience of audiovisual stimuli, so that multisensory stimuli are more resistant to the attenuation of their perceptual salience due to IOR. Therefore, the audiovisual IOR effect was significantly smaller than the visual IOR. The suggestion presents novel insights into the interplay of attention and audiovisual integration.

ERP results revealed that IOR effects on the audiovisual objects ERPs consisted mainly of the increased P1 amplitudes, followed by decreased N1 and P2 effect for valid cue objects. Previous studies suggested that the behavioral IOR effect observed is determined by the contributing effect exerted by a range of components: perceptual (P1) (Prime and Jolicoeur, 2009; Satel et al., 2013; Martín-Arévalo et al., 2014), late-perceptual (N1, Nd) (Wascher and Tipper, 2004; Gutiérrez-Domínguez et al., 2014), spatial selection (N2pc) (Pierce et al., 2017), and decision processes (P3) (Prime and Jolicoeur, 2009). Our results showed that the early positive component (P1) was noticeably greater in valid cue as compared with that in invalid cue condition and followed by a negative effect (N1) was reversed, which was obviously greater in invalid cue as compared with that in valid cue condition. Components of IOR are likely to have the operation in various phases from time distribution (Ivanoff and Klein, 2006). Most existing studies showing the IOR effect were correlated with decreased perceptual sensitivity. For example, the IOR effect is extensively related to the decrease of the early component amplitude (P1, N1) in the valid cue in contrast with invalid cue location independent of the task at hand (Prime et al., 2006; Chica and Lupiáñez, 2009; Prime and Jolicoeur, 2009; Satel et al., 2013, 2014; Martín-Arévalo et al., 2014). However, according to a number of researchers, cueing effects (either facilitation or IOR) reflect the modulation of multiple stages of processing (Hunt and Kingstone, 2003; Berlucchi, 2006; Lupiáñez, 2010), although no agreement has been reached about which is (or are) not the key neural marker of the IOR with the audiovisual target. The attentional bias may be associated with the decrease of the N1 component in location attended, which reflects the behavioral IOR effect. However, according to the recent review (Martínarévalo et al., 2015), there is not a definitive correspondence between the P1 modulation for the valid cue in contrast with invalid cue location trial. It deserves further electrophysiological studies.

### Audiovisual Integration of Audiovisual Stimuli

One of the behavioral effects of the audiovisual integration is that audiovisual stimuli elicit more rapid and precise responses in contrast to the auditory or visual modality alone (Hershenson, 1962; Miller, 1982; Frassinetti et al., 2002). In this study, the behavioral results showed that the ACC was higher than 95% for all participants. Importantly, the RTs for audiovisual dual-modal targets were significantly faster than those for unimodal visual or auditory targets, indicating the redundant signal effects, which is consistent with the previous findings (Hershenson, 1962; Talsma and Woldorff, 2005). Besides behavioral effects, the neural activities of differences between AV targets and the sum of the A and V targets are also calculated to present the audiovisual integration effect (Giard and Peronnet, 1999; Molholm et al., 2002). Specifically, we observed several phases of audiovisual integration effects in ERP results. The earliest of these integration effects was observed during 60∼90 ms at frontal-central-parietal electrodes only in invalid cue conditions, which was followed by three phases of audiovisual integration, regardless of different cue validity. The mentioned comprised a centro-medial negative beginning at nearly 120 ms post-stimulus, followed by a positive wave during 200∼260 ms after stimulus onset. In addition, a positive slow wave started around 340 ms. The identified scalp distribution effect comply with audiovisual integration effect under the description previously (Giard and Peronnet, 1999; Fort et al., 2002). Previous studies showed that when attention is directed to both modalities simultaneously, audiovisual stimuli can integrate very early in the sensory flow of processing (∼50 ms post-stimulus). Attention critically helps initiate the mentioned early audiovisual stimuli integration. In our study, participants were asked to distribute their attention to different forms; thus, we observed the early effects of audiovisual integration within 100 ms.

### Moderating Influence of Inhibition of Return on Integration

To our knowledge, the neural underpinning modulation of audiovisual integration by IOR has been rarely studied. As noted in introduction, the differences in unimodal signal strength hypothesis have been developed to explain this modulation but only based on the behavioral evidence. In this work, we utilized the ERP technique to explore the neural underpinnings of this modulation. The results seem to conflict with the assumption of unimodal differences in unisensory processing. The main point of differences in unimodal signal strength hypothesis is as follow: when there are large differences in signal strength between different sensory modalities, AVI effects are smaller; and when the signal strength is similar between different sensory modalities, AVI effects are larger. The unimodal V/A input, which is influenced by the IOR, is reflected in the signal strength of AV target stimuli. Here, we compared the difference between the absolute value of A and V between the valid (*M* = 1.22, *SD* = 0.67) and invalid cue condition (*M* = 0.95, *SD* = 1.01), the results showed that the difference between these two conditions was not significant, *t* (20) = 1.1, *p* = 0.28. Then, the relationship between the size of the differences in unimodal value and audiovisual integration effects on P70 effect was evaluated using the Pearson correlation. But there was also no significant correlation between them (*r* = 0.37, *p* = 0.1). Therefore, the results were not supportive of the hypothesis of differences in unimodal signal strength.

In addition, Van der Stoep et al. (2015) have proposed a perceptual sensitivity hypothesis to explain the modulation mechanisms of exogenous spatial attention to the audiovisual integration. In this study, researchers examined impacts exerted by facilitation effects induced by exogenous auditory cues with short SOA (200∼250 ms) on audiovisual integration. The same result was found in this study where exogenous spatial attention reduced the audiovisual integration effects at valid cue locations. Researchers considered the exogenous cues improve the perceptual sensitivity at the valid cue location and further increase the perceptual sensitivity of the targets at that location (Carrasco, 2011). According to the principle of inverse effectiveness (Meredith and Stein, 1983; Holmes, 2007), audiovisual integration benefit turns out to be more pronounced for relatively weak stimuli as compared with relatively strong stimuli. Therefore, audiovisual integration can be reduced at valid cue locations when exogenous spatial attention can increase perceptual sensitivity at valid cue locations which abides by inverse effectiveness principles. In our work, the ERP results supported the perceptual sensitivity explanation. Specifically, we observed the early positive component (P1) is enhanced at valid cue conditions compared to invalid cue conditions, which can increase the contrast sensitivity at valid cue locations (Carrasco, 2011). Therefore, audiovisual integration would be reduced at the valid cue location in contrast with the invalid cue locations. Importantly, early audiovisual integration ERP effects were found in the P70 component (60∼90 ms, frontal-central-parietal) only under the invalid cue condition. The mentioned observation follows the perspective that only low-intensity stimuli can induce the early (40∼60 ms) audiovisual integration effect (Senkowski et al., 2011).

As discussed above, our data provide clear physiological evidence for the assertion that IOR can impact the processes involved in the integration of audiovisual stimuli. Our study conducted the successful replication of an early integration effect (the P70 component). This early audiovisual integration process occurred early and indicated inside ERPs in particular circumstance: when both the audiovisual senses were fully attended. Importantly, IOR effect was suggested to decrease early audiovisual integration amplitude at valid cue locations than to those at invalid cue around 60 ms after stimulus onset. Notably, although the behavioral effects of exogenous spatial cueing on audiovisual integration are similar for short (facilitation effect) and long SOAs (IOR; this study), the underlying neural mechanisms may be different. This prospect deserves further research.

## Conclusion

We used a cue-target paradigm to investigate the neural underpinnings of IOR for audiovisual stimuli at long SOAs (400–600 ms). We found that IOR modulated the early audiovisual integration (P70 component). Specifically, audiovisual integration decreased at valid cue location in contrast to invalid cue location under the focus on several modalities, supporting the assumption of perceptual sensitivity. This work offers initial neural evidence that IOR decreases early audiovisual integration.

## Data Availability Statement

The original contributions presented in the study are included in the article/supplementary material, further inquiries can be directed to the corresponding author/s.

## Ethics Statement

The studies involving human participants were reviewed and approved by the Ethics Committee at the Civil Aviation Flight University of China. The patients/participants provided their written informed consent to participate in this study.

## Author Contributions

XT, RC, and MZ contributed to conception and design of the study. XP and HJ organized the database. XP and AW performed the statistical analysis. XP wrote the first draft of the manuscript. All authors contributed to manuscript revision, read, and approved the submitted version.

## Conflict of Interest

The authors declare that the research was conducted in the absence of any commercial or financial relationships that could be construed as a potential conflict of interest.

## Publisher’s Note

All claims expressed in this article are solely those of the authors and do not necessarily represent those of their affiliated organizations, or those of the publisher, the editors and the reviewers. Any product that may be evaluated in this article, or claim that may be made by its manufacturer, is not guaranteed or endorsed by the publisher.
